# Tumor suppressor role of microRNA-1296 in triple-negative breast cancer

**DOI:** 10.18632/oncotarget.6961

**Published:** 2016-01-20

**Authors:** Binh Phan, Shahana Majid, Sarah Ursu, David de Semir, Mehdi Nosrati, Vladimir Bezrookove, Mohammed Kashani-Sabet, Altaf A. Dar

**Affiliations:** ^1^ California Pacific Medical Center Research Institute, San Francisco, CA 94107, USA; ^2^ Department of Urology, Veterans Affairs Medical Center and University of California San Francisco, San Francisco, CA 94121, USA

**Keywords:** microRNA-1296, triple negative breast cancer, tumor suppressor, cyclin D1, apoptosis

## Abstract

Triple negative breast cancer (TNBC) is an aggressive subtype of breast cancer with a poor prognosis, which lacks effective targeted therapies. There is an urgent need to better understand the underlying molecular mechanisms of TNBC aggressiveness and identify novel, efficient targets for therapeutic intervention.

**Methods:**

miRNA qRT-PCR was used to determine the expression of miR-1296 in cell lines. The miR-1296 overexpression effects in TNBC cell lines were investigated using assays of colony formation, cell cycle and apoptosis. Immunoblotting was performed to determine the expression of the miR-1296 target protein, and luciferase assays were performed to confirm the target of miR-1296 action.

**Results:**

miR-1296 expression was significantly suppressed in TNBC cell lines and tissues samples. Overexpression of miR-1296 significantly suppressed cell proliferation of two TNBC cell lines when compared to control miRNA-expressing cells. A significant decrease in the S-phase of the cell cycle was observed following miR-1296 overexpression, accompanied by induction of apoptosis in TNBC cells. Cyclin D1 (CCND1) was identified as a target of miR-1296 action. miR-1296 overexpression significantly suppressed the luciferase activity of reporter plasmid containing the 3′UTR of CCND1 and protein expression levels of CCND1 in TNBC cells. The effects of miR-1296 overexpression on TNBC cell growth were reversed by CCND1 overexpression. miR-1296 expression sensitized TNBC cells to cisplatin treatment.

**Conclusion:**

Our results demonstrate a novel tumor suppressor role for miR-1296 in triple-negative breast cancer cell lines, identify CCND1 as its target of action, and demonstrate a potential role for miR-1296 in sensitizing breast cancer cells to cisplatin.

## INTRODUCTION

Triple-negative breast cancer (TNBC) is an invasive carcinoma of the breast that lacks expression of the estrogen receptor (ER), progesterone receptor (PR) and human epidermal growth factor receptor 2 (HER2) [1, 2]. TNBC accounts for about 12-17% of all breast cancers, and is often a highly invasive and aggressive form of breast cancer, with very poor prognosis compared with other subtypes. Current treatment modalities for TNBC are limited to surgery, radiation and systemic chemotherapy due to the lack of more specific therapeutic targets. In patients who respond to chemotherapy, the remission period is relatively short, and progression is expected within a short period of time [3, 4]. There is an urgent need to better understand the underlying molecular mechanisms of TNBC aggressiveness and identify novel, efficient targets for therapeutic intervention. Significant effort has been expended in the last several years in search of a molecular targeted therapy for TNBC. However, to date, no successful targeted therapies exist for advanced stages of TNBC [5].

MicroRNAs (miRNA) are a large class of small, non-coding RNA molecules involved in gene regulation through binding to the 3′ untranslated region (3′UTR) of their target mRNAs, resulting in mRNA degradation or translation inhibition [6, 7]. miRNAs are expressed in a tissue-specific manner and are central regulators of gene expression. They can act either as oncomirs by targeting tumor suppressors, or as tumor suppressors by targeting oncogenes [6, 8]. Inactivation of oncogenic miRNAs [9, 10] or restoration of tumor-suppressor miRNAs [11, 12] may have great potential for cancer treatment. miRNAs are found to be critically involved in many fundamental processes of cancer [7, 13], although the underlying mechanisms have not been well understood for the majority of miRNAs. They have been reported to be encoded in cancer-related regions (regions of loss of heterozygosity, minimal regions of amplification or common breakpoint regions), suggesting that changes in miRNA expression might have a causal relationship to tumorigenesis [14, 15]. Due to their tremendous regulatory potential and tissue-specific and disease-specific expression patterns, there is increasing evidence that miRNA expression profiles could be indicative of disease risk or burden [16]. In breast cancer, miRNAs are shown to affect cancer cell survival, proliferation, differentiation, migration, invasion and metastasis [17-19]. However, few studies on the role of miRNAs in TNBC have been performed when compared with other breast cancer subtypes. Thus, studying the role of individual miRNAs in TNBC may lead to identification of novel therapeutic targets.

CCND1 plays a key role in regulation of the G1-S phase transition and in tumorigenesis [20]. Expression of CCND1 is induced by a broad array of oncogenic stimuli and is required for contact-independent growth [20, 21]. CCND1 is a well-characterized oncogene that is frequently overexpressed in human breast, lung, colon, prostate and hematopoietic carcinomas [22, 23], and its overexpression is frequently cited as a potential biomarker [24, 25]. However, the underlying mechanisms of CCND1 overexpression and its connection to breast cancer progression are poorly understood.

This report describes, for the first time, a functional role for miR-1296 in triple negative breast cancer, identifies CCND1 as a target of miR-1296 action, and demonstrates the potential for miR-1296 to sensitize TNBC cells to chemotherapeutic agents.

## RESULTS

### Expression of miR-1296 in triple negative breast cancer cell lines and tissues, and effects of its overexpression

**Figure 1:**
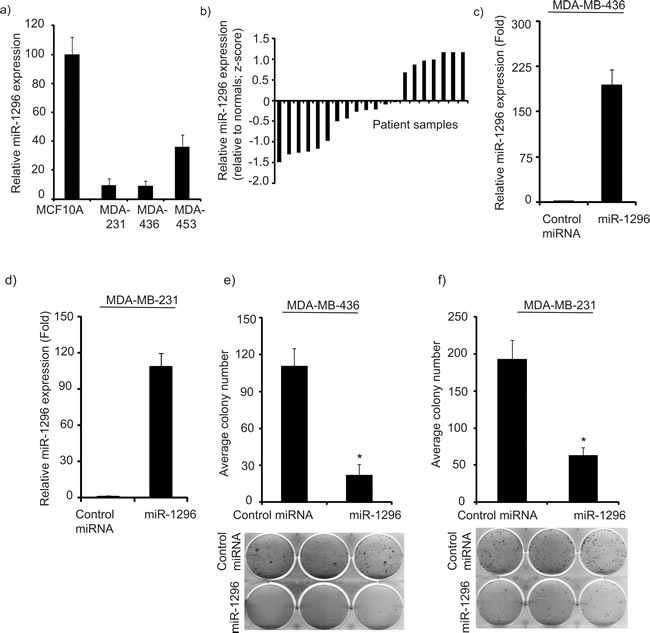
miR-1296 is suppressed in triple negative breast cancer cell lines and tissues **a.** miR-1296 expression is significantly suppressed in triple negative breast cancer cell lines when compared to non-tumorigenic breast cell line (MCF10A). **b.** miR-1296 expression is suppressed in a majority of triple negative breast cancer samples when compared to normal breast samples. **c.-d.** Relative miR-1296 expression levels in 436 and 231 cells following miR-1296 transfection, as determined by miR qRT-PCR. **e.-f.**) miR-1296 overexpression reduced the colony formation ability of 436 and 231 cells, respectively when compared to control miRNA-expressing cells. *p < 0.05.

We analyzed expression of miR-1296 in non-tumorigenic MCF10A cells and in a panel of triple negative breast cancer (TNBC) cells: MDA-MB-231, MDA-MB-436 and MBA-MB-453. miR-1296 was significantly suppressed in all three TNBC cell lines when compared to non-tumorigenic MCF-10A cells as determined by miRNA qRT-PCR analysis (Figure 1a). miR-1296 expression from the TCGA database was available for 20 triple negative tumors and 11 normal breast samples. miR-1296 expression was suppressed in 13/20 (65%) TNBC samples as compared to normal breast samples (Figure 1b). These observations suggested that miR-1296 might have a potential tumor suppressive role in triple negative breast cancer.

Transient transfection of miR-1296 (Figure 1c-1d) into TNBC cells significantly suppressed cell survival (by 80% to 69%) when compared to cells expressing control miRNA (Figure 1e-1f) in 436 and 231 cells, respectively. miR-1296-overexpressing TNBC cells showed a reduction in the number as well as in size of foci when compared to control miR-expressing cells.

### miR-1296 overexpression regulates cell proliferation and cell cycle

**Figure 2:**
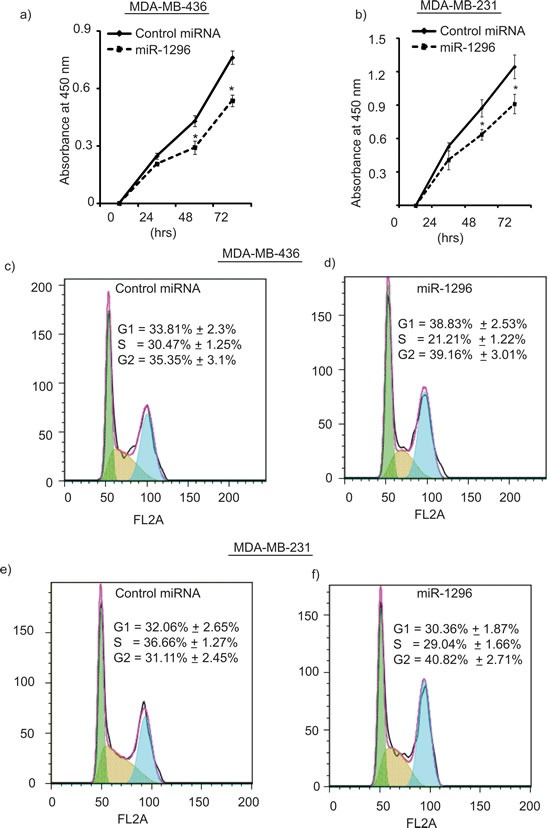
miR-1296 regulates cell proliferation and cell cycle phases **a.-b.** miR-1296 overexpression suppresses cell proliferation in 436 and 231 cells, respectively, as compared to control miRNA-expressing cells. **c.-d.** miR-1296 overexpression in 436 cells suppresses S-phase as compared to control miRNA-expressing cells. **e.-f.** miR-1296 overexpression reduces S-phase of 231 cells compared to control miRNA-expressing cells. *p < 0.05.

miR-1296 overexpression in TNBC cells significantly suppressed cell proliferation when compared to control miRNA expressing cells (Figure 2a-2b) as determined by the Cell Counting Kit. Overexpression of miR-1296 in TNBC cells had a significant impact on the cell cycle. A significant decrease in the S-phase (from 30.47 % to 21.21%) of 436 cells overexpressing miR-1296 was observed when compared to control miR-expressing cells (Figure 2c-2d). Furthermore, miR-1296 overexpression led to G2/M arrest in 436 cells. To confirm the effects of miR-1296 overexpression on the cell cycle, miR-1296 was transfected into 231 cells, and a significant decrease in S-phase was observed, along with G2/M arrest (Figure 2e-2f). These results indicate that miR-1296 overexpression regulates the different phases of the cell cycle in triple negative breast cancer cells and thus affects their proliferation pattern.

### Overexpression of miR-1296 induces apoptosis in TNBC

**Figure 3:**
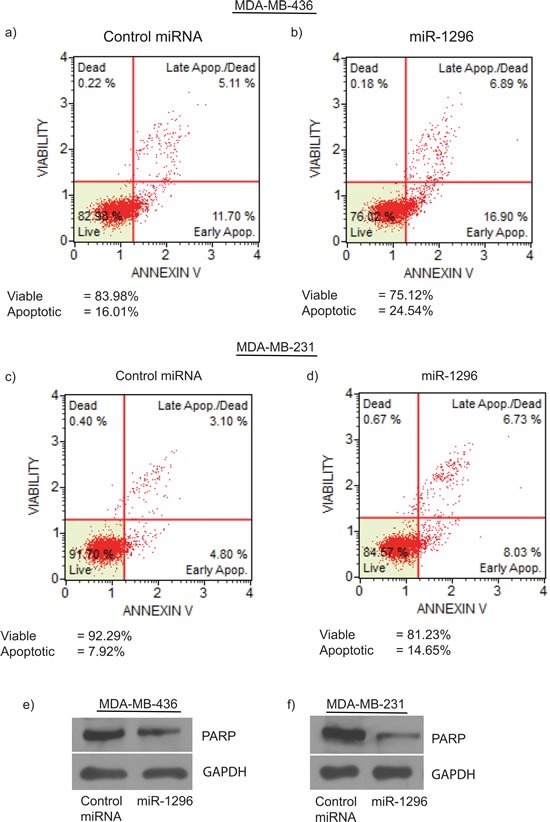
miR-1296 induces apoptosis miR-1296 overexpression in 436 and 231 cells, respectively, induced apoptosis **b.** and **d.** as compared to control miRNA-expressing cells **a.** and **c. e.-f.** miR-1296 overexpression enhances PARP cleavage in 436 and 231 cells, respectively.

miR-1296 overexpression in 436 cells substantially induced apoptosis (from 16.01% to 24.54%) with a concomitant decrease in living cells when compared to control miR-expressing cells (Figure 3a-3b). To confirm the induction of apoptosis by miR-1296 overexpression, similar results were observed in another triple negative breast cancer cell line (231) following miR-1296 overexpression (Figure 3c-3d). miR-1296 overexpression affected the apoptotic marker PARP by enhancing its cleavage to induce apoptosis in 436 and 231 cells (Figure 3e-3f), respectively.

### CCND1 as a target of miR-1296

**Figure 4:**
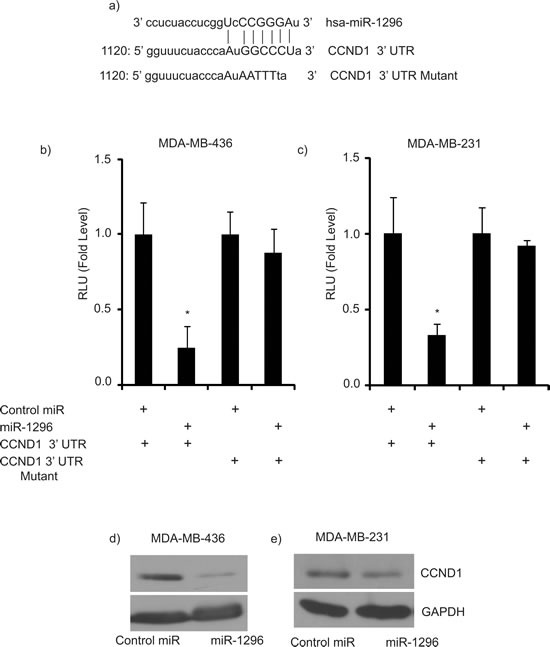
CCND1 as target of miR-1296 **a.** The seed sequence of miR-1296 is complementary to the 3′UTR of CCND1. **b.-c.** Luciferase assay showing reduction in reporter activity (relative luciferase units) after co-transfection of CCND1-3′UTR with miR-1296 in 436 and 231 cells, respectively. The mutant 3′UTR had no effect on reporter activity. **d.-e.** Western blot analysis showing suppression of CCND1 protein levels in 436 and 231 cells after miR-1296 overexpression, respectively. *p < 0.05.

**Figure 5:**
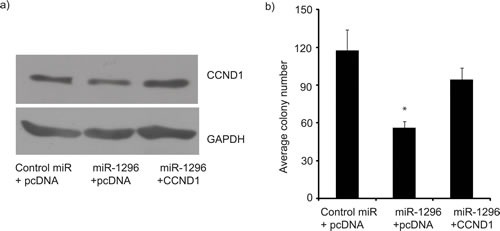
CCND1 overexpression reverses miR-1296 effects Co-transfection of a CCND1-expressing vector, along with miR-1296, restored CCND1 expression (panel a) and reversed the suppression of colony formation ability (panel b) induced by miR-1296 expression in 436 cells. *p < 0.05.

To identify potential effectors of miR-1296 action, we used algorithms and sequence alignments that predict miRNA targets, and identified CCND1 as a putative target, as the seed sequence of miR-1296 was complementary to the 3′UTR of CCND1 (Figure 4a). To confirm CCND1 as a direct target of action of miR-1296, the 3′UTR of CCND1 harboring the complementary sequence to the miR-1296 seed sequence was cloned in a luciferase reporter plasmid vector. Transient co-transfection of the CCND1-3′UTR construct along with miR-1296 into 436 and 231 TNBC cells led to a significant decrease in reporter expression when compared with the control 3′ UTR vector, whereas transfection of a mutant CCND1 3′UTR sequence not complementary to miR-1296 abolished the suppressive effect on reporter expression (Figure 4b-4c). These results indicate that the conserved nucleotides in the 3′UTR of CCND1 are responsible for miR-1296 targeting *in vitro*. Transient miR-1296 overexpression significantly suppressed CCND1 expression at the protein level, confirming that miR-1296 regulates CCND1 expression in 436 and 231 TNBC cells (Figure 4d-4e). To further explore the role of CCND1 as a target of miR-1296, we co-transfected 436 cells with miR-1296, along with a vector encoding CCND1 cDNA, and examined effects on gene expression and cell survival. Co-transfection of miR-1296 and an empty vector control resulted in suppression of CCND1 and suppressed cell survival (Figure 5a-5b). These effects were largely reversed following co-transfection of miR-1296 and the CCND1-expressing vector (Figure 5a-5b), indicating that the effects of miR-1296 on gene expression and cell proliferation are mediated largely by its inhibition of CCND1 expression.

### miR-1296 sensitizes cells to cisplatin treatment

**Figure 6:**
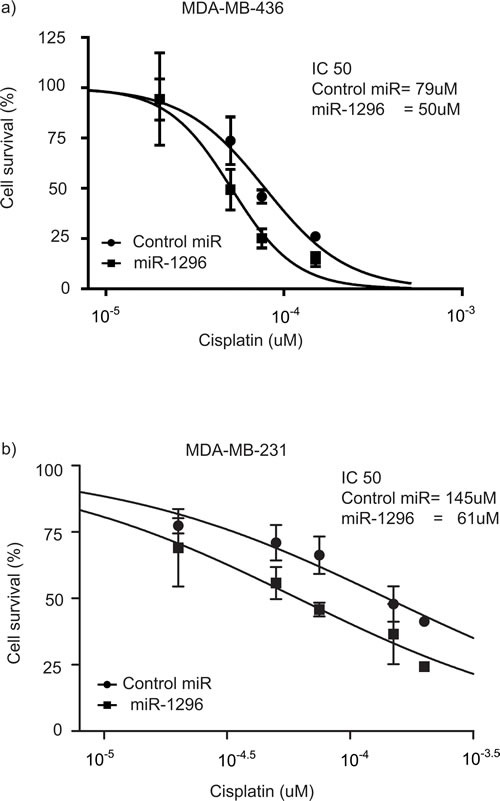
miR-1296 sensitizes cells to chemotherapy drugs **a.-b.** Overexpression of miR-1296 in 436 and 231 cells, respectively, significantly sensitized cells to cisplatin treatment as compared to control miRNA. Cells were treated with cisplatin 24 hr following miR-1296 transfection and the cell viability was assessed after 48 hr of cisplatin treatment.

Platinum-based compounds have recently shown promising activity in the setting of triple negative breast cancer [26]. Given that our results indicate that miR-1296 overexpression significantly regulated cell cycle and induced apoptosis in TNBC cells, we investigated whether miR-1296 expression could modulate sensitivity of TNBC cells to chemotherapy drug like cisplatin. TNBC cells were transfected with miR-1296, and 24hr later the cells were treated with varying concentrations of cisplatin. Cell viability was assessed after 48hr of cisplatin treatment by a Cell Counting Kit. As shown in Figure 6a-6b, miR-1296 overexpression sensitized 436 and 231 cells to cisplatin treatment (by 1.56- or 2.4- fold, respectively).

## DISCUSSION

Triple negative breast cancers are a heterogeneous subset of tumors grouped together based on their lack of hormone receptor and HER2 expression. The clinical relevance of triple negative breast cancers is exemplified by the relatively poor prognosis and lack of effective treatments. There is an urgent clinical need to identify novel agents and safe and effective treatments for triple-negative breast cancer. Altered miRNA expression has been shown to be a common event that can control cell proliferation in the progression of breast cancer [27, 28]. Deregulation of miRNAs in cancer cells and their roles in tumorigenesis have been increasingly investigated. However, the role of miR-1296 and its biological significance has not been studied yet. miR-1296 is a largely unstudied miRNA, other than a previous study in prostate cancer [29]. As a result, little is known of its functional role in cancer.

In the present study, we examined the functional significance of miR-1296 in TNBC, along with its regulation of CCND1. Here, we report suppression of miR-1296 expression in TNBC cells, and demonstrate its role as a tumor suppressor. miR-1296 is downregulated in TNBC cells when compared to a non-tumorigenic breast cell line, and in the majority of human triple negative breast cancer samples when compared to normal breast samples *In silico* algorithms and sequence alignments identified the CCND1 oncogene as a potential target. Our results demonstrated that miR-1296 directly targets the 3′UTR of CCND1, as its overexpression was associated with suppression of luciferase activity in a reporter plasmid. In addition, a significant downregulation of CCND1 protein levels was observed following miR-1296 overexpression, indicating the post-transcriptional regulation of CCND1 via targeting its 3′UTR. CCND1 is a well-characterized oncogene that is frequently overexpressed in many tumors [22, 23]. Overexpression of CCND1 is tumorigenic, as supported by evidence that MMTV-driven CCND1 overexpression is sufficient for mammary hyperplasia and carcinoma development in transgenic mice [30]. CCND1 overexpression is a common event in cancer, and is usually a result of defective regulation at the post-translational level [31, 32]. Therefore, regulation of CCND1 at protein level can play a critical role in tumor development. We demonstrated suppression of CCND1 at the protein level following miR-1296 overexpression, thereby making it a critical agent to regulate CCND1 post-translationally. miR-1296 overexpression substantially decreased cell proliferation and survival of TNBC cells. To confirm that miR-1296 suppresses tumor cell growth due to CCND1 regulation, we found that CCND1 cDNA overexpression could rescue the growth suppression induced by miR-1296 overexpression alone. These results indicate that miR-1296 inhibits TNBC cell growth and proliferation, at least in part, by targeting CCND1. To date, a few other miRNAs have been reported to regulate TNBC cell growth, invasiveness, migration and metastasis [33-35]. CCND1 is activated in many cancers; this has prompted much focus on the development of anti- CCND1-based therapy [36]. Recent findings indicate that CCND1 regulates transcription factors, histone acetylation, cellular metabolism and cell migration [22, 36], all of which contribute to tumorigenesis. Regulating CCND1 expression represents an alternative approach rather than the conventional strategy of developing small molecule CDK inhibitors. Our results identify miR-1296-based suppression of CCND1 as a novel targeted approach for the therapy of TNBC. Furthermore, miR-1296 overexpression sensitized TNBC cells to cisplatin treatment. Platinum-based treatments alone or in combination have generated interest in treating TNBC [37], due to lack of treatment options for this subtype of breast cancer, and their use has been supported by the strong association of TNBC tumors with germline mutations in the BRCA1 gene. TNBC patients have shown better survival rates in response to cisplatin treatment, though the development of acquired resistance is a significant obstacle for this treatment [26]. Cisplatin-induced breast cancer cell death is associated with a decrease in the expression levels of CCND1 [38]. Our results show that miR-1296 suppresses CCND1, thus, miR-1296 alone or in combination with cisplatin, might be an alternative approach to target TNBC to improve the overall outcome of TNBC patients. Further studies of the effects of miR-1296 expression on cisplatin cytotoxicity will be required to confirm the clinical rationale for this approach.

Our study demonstrates that miR-1296 is suppressed in TNBC cells and tissues. miR-1296 overexpression results in downregulation of CCND1, suppression of the proliferative ability of TNBC cells, and induction of tumor cell apoptosis. miR-1296 overexpression can sensitize cells to cisplatin treatment. Overall, these studies describe a novel tumor suppressor role for miR-1296 in TNBC cells, and suggest a potential therapeutic role in combination with platinum-based drugs.

## MATERIALS AND METHODS

### Cell culture, plasmids and transfection

Triple negative breast cancer (TNBC) cell lines MDA-MB-231, MDA-MB-436 and MDA-MB-453, as well as MCF10A cells were purchased from ATCC (ATCC, Manassas VA). TNBC cells were grown in RPMI with 5% fetal bovine serum, whereas MCF10A was grown in DMEM/F12 supplemented with serum horse, cholera toxin, epidermal growth factor, hydrocortisone and insulin. All cells were cultivated at 37°C in an atmosphere containing 5% CO_2_. MDA-MB-231 will be referred to as 231 and MDA-MB-436 as 436 in the text, respectively. 231 and 436 cells belong to the basal subtype (ER^−^/PR^−^/HER2^−^) of breast cancer, obtained from pleural effusions. 231 are aneuploid with a modal number of 64, whereas, for 436 the modal number is 45. Plasmids pcDNA3.1 (Life Technologies, Carlsbad, CA), pcDNA-CCND1 (Addgene, Cambridge MA) and miRNA target vector pmirGLO-dual luciferase miRNA expression vector (Promega, Madison, WI) were purchased. miRNA precursors for miR-1296 and controls were purchased from Life Technologies (Carlsbad, CA). Transient transfections were carried out by Lipofectamine-2000 (Life Technologies, Carlsbad, CA) according to the manufacturer's protocol. Cisplatin was purchased from Sigma-Aldrich (St. Louis, MO)

### miRNA extraction and quantitative real-time PCR

miRNAs were extracted by using the mirVana miRNA extraction kit (Life Technologies, Carlsbad, CA) from cell lines following the manufacturer's instructions. Mature miRNAs were assayed using the TaqMan MicroRNA Assays, in accordance with the manufacturer's instructions (Life Technologies, Carlsbad, CA). All RT reactions, including no-template controls and RT minus controls, were run in a 7500 Fast real time PCR system (Life Technologies, Carlsbad, CA). RNA concentrations were determined with a NanoDrop (Thermo Scientific, Rockford, IL). Samples were normalized to RNU48 for miRNAs (Life Technologies, Carlsbad, CA) as indicated. Gene expression levels were quantified using the 7500 Fast real time sequence detection system software (Life Technologies, Carlsbad, CA). Comparative real-time PCR was performed in triplicate, including no-template controls. Relative expression was calculated using the comparative Ct method. miR-1296 expression for triple negative breast cancer samples (*n* = 20) and normal breast cancer samples (*n* = 11) was obtained from the publically available TCGA database. Expression of miR-1296 is reported as a z-score relative to normal samples.

### Cell proliferation and colony formation assays

Cell proliferation was assessed at 24, 48 and 72 hr post-transfection of miR-1296 using Cell Counting Kit-8 (Dojindo, Rockville MD) following the manufacturer's protocol. For the colony formation assay, 200 cells were plated in a 6-well plate in triplicate, and allowed to grow until visible colonies appeared. Colonies were stained with Giemsa and counted.

### Western blot analysis

Cell lysates were prepared in phosphate buffer saline containing 1x Halt protease inhibitor cocktail and 1x Halt phosphatase inhibitor cocktail (Pierce, Rockford, IL) centrifuged at 3500 r.p.m. for 10 min at 4°C. Proteins (10-15 ug) from each sample were subjected to SDS/polyacrylamide gel electrophoresis and transferred onto a nitrocellulose membrane. Target proteins were detected by using specific antibodies against GAPDH (Cell Signaling Technology, Danvers, MA), PARP and CCND1 (Santa Cruz Biotechnology, Santa Cruz, CA).

### Luciferase assays

The 3′-UTR region of CCND1 containing target site sequences complementary to the seed sequence of miR-1296 was cloned downstream of the luciferase gene in the pmiR-Glo dual luciferase vector (Promega, Madison, WI), and the resultant vector named CCND1-3′UTR. The sequence of the oligonucleotides cloned is (5′ gguuucuacccaAuGGCCCUa 3′). Mutated 3′UTR sequences of CCND1 complementary to miR-1296 were cloned in the same vector and the resultant vector named CCND1-3′UTR Mutant. The mutated sequence of the oligonucleotides cloned is (5′ gguuucuacccaAuAATTTta 3′). Firefly luciferase activity was measured by using the Dual Luciferase Assay (Promega, Madison, WI) 24 hr after transfection and the results were normalized with Renilla luciferase. Each reporter plasmid was transfected at least three times and each sample was assayed in triplicate.

### Cisplatin treatment

TNBC cells were treated with different concentrations of cisplatin 24 hr following miR-1296 transfection, as indicated. Cell viability was assessed after 48 hr of cisplatin treatment using Cell Counting Kit-8 following the manufacturer's instructions.

### Apoptosis and cell cycle analysis

For apoptosis and cell cycle, Muse^®^ Annexin V and Dead Cell Assay Kit and Muse^®^ Cell Cycle Assay Kit (EMD Millipore, Billerica MA) were used respectively, as per the manufacturer's instructions. Briefly, for cell cycle analysis cells were trypsinized, washed with PBS and fixed in 70% ethanol overnight. Cells were centrifuged, washed with PBS, dissolved in 200ul of Muse cell cycle assay kit, and analyzed by Muse Cell Analyzer.

### Statistical analysis

All quantified data represent an average of at least triplicate samples or as indicated. Error bars represent standard error of the mean. Statistical significance was determined by the Student's *t*-test and two-tailed *p* values <0.05 were considered significant. For cisplatin sensitivity, data was analyzed by Graphpad Prism 6 (La Jolla, CA).
